# Music Upper Limb Therapy—Integrated: An Enriched Collaborative Approach for Stroke Rehabilitation

**DOI:** 10.3389/fnhum.2016.00498

**Published:** 2016-10-07

**Authors:** Preeti Raghavan, Daniel Geller, Nina Guerrero, Viswanath Aluru, Joseph P. Eimicke, Jeanne A. Teresi, Gbenga Ogedegbe, Anna Palumbo, Alan Turry

**Affiliations:** ^1^Department of Rehabilitation Medicine, New York University School of MedicineNew York, NY, USA; ^2^Steinhardt School of Culture, Education, and Human Development, New York UniversityNew York, NY, USA; ^3^Research Division, Hebrew Home at RiverdaleBronx, NY, USA; ^4^Division of Geriatrics and Palliative Medicine, Weill Cornell Medical CollegeNew York, NY, USA; ^5^Columbia University Stroud Center and New York State Psychiatric InstituteNew York, NY, USA; ^6^Department of Population Health, New York University School of MedicineNew York, NY, USA

**Keywords:** rehabilitation, functional recovery, music therapy, enriched environment, bodily perception, social participation, psycho-social adjustment, sense of self

## Abstract

Stroke is a leading cause of disability worldwide. It leads to a sudden and overwhelming disruption in one’s physical body, and alters the stroke survivors’ sense of self. Long-term recovery requires that bodily perception, social participation and sense of self are restored; this is challenging to achieve, particularly with a single intervention. However, rhythmic synchronization of movement to external stimuli facilitates sensorimotor coupling for movement recovery, enhances emotional engagement and has positive effects on interpersonal relationships. In this proof-of-concept study, we designed a group music-making intervention, Music Upper Limb Therapy-Integrated (MULT-I), to address the physical, psychological and social domains of rehabilitation simultaneously, and investigated its effects on long-term post-stroke upper limb recovery. The study used a mixed-method pre-post design with 1-year follow up. Thirteen subjects completed the 45-min intervention twice a week for 6 weeks. The primary outcome was reduced upper limb motor impairment on the Fugl-Meyer Scale (FMS). Secondary outcomes included sensory impairment (two-point discrimination test), activity limitation (Modified Rankin Scale, MRS), well-being (WHO well-being index), and participation (Stroke Impact Scale, SIS). Repeated measures analysis of variance (ANOVA) was used to test for differences between pre- and post-intervention, and 1-year follow up scores. Significant improvement was found in upper limb motor impairment, sensory impairment, activity limitation and well-being immediately post-intervention that persisted at 1 year. Activities of daily living and social participation improved only from post-intervention to 1-year follow up. The improvement in upper limb motor impairment was more pronounced in a subset of lower functioning individuals as determined by their pre-intervention wrist range of motion. Qualitatively, subjects reported new feelings of ownership of their impaired limb, more spontaneous movement, and enhanced emotional engagement. The results suggest that the MULT-I intervention may help stroke survivors re-create their sense of self by integrating sensorimotor, emotional and interoceptive information and facilitate long-term recovery across multiple domains of disability, even in the chronic stage post-stroke. Randomized controlled trials are warranted to confirm the efficacy of this approach. Clinical Trial Registration: National Institutes of Health, clinicaltrials.gov, NCT01586221.

## Introduction

Stroke affects one in six individuals worldwide, and is the leading cause of disability (Thrift et al., 2014). In the vast majority of survivors, the sudden and lasting physical effects of stroke lead to a catastrophic disruption in their sense of self and in relationships with the physical and social world (Ellis-Hill and Horn, 2000; Ellis-Hill et al., 2000; Secrest and Zeller, 2007; Salter et al., 2008). Depressed mood, social isolation, poor subjective well-being and mental distress contribute to increased motor impairment, disability and risk of future stroke (Ostir et al., 2001; Northcott et al., 2015). Long-term recovery is thought to be strongly influenced by coherence between the stroke survivor’s bodily perception, participation in everyday life, and sense of self (Arntzen et al., 2015). Traditional multi-disciplinary rehabilitation addresses physical limitations such as immobility and reduced functional independence, psychological limitations such as depressed mood and lack of motivation, and societal limitations such as social isolation one at a time. For example, patients may receive physical therapy for a few weeks, and subsequently or separately receive cognitive therapy or psychotherapy. Each type of therapy leads to changes in network connectivity between specific regions of the brain depending on the information that is processed during the therapy tasks (Bajaj et al., 2015a). In contrast, combination therapies can increase the connectivity between multiple brain regions that are disconnected after stroke, leading to better functional outcomes (Bajaj et al., 2015b). Since multi-disciplinary rehabilitation is not widely available—only 30% of individuals who need rehabilitation actually receive it (Go et al., 2013), and there are increasing disparities in accessibility to rehabilitation in the chronic post-stroke period (Roth et al., 2011; Winstein et al., 2016)—combination therapies may be the solution to address limitations across multiple domains simultaneously. Here we asked if a single combined intervention could be designed to address physical, psychological and social domains of rehabilitation simultaneously to facilitate long-term post-stroke upper limb recovery.

Music is one of the most powerful elicitors of spontaneous motor actions (Jäncke, 2008). It motivates people to adhere to exercise regimens (Wininger and Pargman, 2003), distracts attention from physical effort, and reduces perceived exertion (Dyrlund and Wininger, 2008). In addition, auditory-motor coupling has been shown to facilitate repetitive movements post-stroke (Roerdink et al., 2009; Rojo et al., 2011; Rodriguez-Fornells et al., 2012), and repetitive and rhythmic movement synchrony between individuals can establish and reinforce social bonds (Hove and Risen, 2009; Miles et al., 2009; Cirelli et al., 2014). Live interactive music-making engages individuals to interact spontaneously and promotes relationship building (Guerrero et al., 2014). Several studies have also shown positive effects of music listening on mood, and on cognitive and motor processing post-stroke (Särkämö et al., 2008; Malcolm et al., 2009; Särkämö and Soto, 2012). Taken together, these studies suggest that music-making activities may be used to integrate physical, psychological and social facets of rehabilitation, creating an enriched environment for post-stroke recovery. Animal studies have shown that in enriched environments the simultaneous physical and mental activity in socially interactive contexts act synergistically to promote neurogenesis, neuronal integration and recovery (Madroñal et al., 2010; Krakauer et al., 2012).

We therefore designed a novel collaborative group music-making intervention, Music Upper Limb Therapy-Integrated (MULT-I), that combined music therapy with occupational therapy to support physical effort, social participation and psychological well-being simultaneously. This study tested the hypothesis that the MULT-I intervention, provided twice a week for 6 weeks, will lead to reduced upper limb motor impairment (primary outcome); and reduced sensory impairment and activity limitation along with increased well-being and participation (secondary outcomes) post-intervention. Since the interaction among physical, psychological and social facets is thought to support long-term recovery, we further hypothesized that the improvement would persist at 1-year follow up.

## Patients and Methods

### Setting and Study Population

Sixteen ethnically diverse subjects with chronic post-stroke hemiparesis were recruited by referral from physicians and therapists from Rusk Rehabilitation, New York University Langone Medical Center and other hospitals in the New York City metropolitan area (Table 1). Inclusion criteria included chronic unilateral stroke at least 6 months prior, the ability to ambulate independently in the community with or without an assistive device, and the ability to grasp implements, such as a wooden mallet, at least partially to participate effectively in the study. Exclusion criteria included hearing deficits that might affect reaction and response to music, as assessed using the Hearing Handicap Inventory for Adults (Newman et al., 1991), history of other neurological or psychiatric disorder, prior injury or surgery in the upper limbs, severe aphasia, cognitive or perceptual deficits including inability to follow directions and attend to task, visual impairment and motor and ideational apraxia or neglect that would prevent participation in the intervention. Subjects did not need to have prior musical training to participate. The New York University Institutional Review Board approved this study (i11-02284) and the subjects gave written informed consent as per the Helsinki Declaration prior to participation in the study. The clinical trial was registered at http://clinicaltrials.gov, NCT01586221.

**Table 1:** **Subject characteristics**.

Subject	FMS (/66)	Age (years)	Ethnic group	Gender	Time since stroke (months)	Handedness/Hemiparesis	Stroke subtype	Lesion location	Amount of OT (months)
1201	17	54	White	M	144	R/R	N/A	Left MCA	8
1237	18	33	Hispanic	F	45	R/L	Hemorrhagic	Right BG/Insula	6
1243	19	49	Black	M	25	R/L	N/A	Right MCA	1
1300	29	21	White	F	48	R/L	Hemorrhagic	Right temporal lobe	2
1257	34	64	White	F	24	R/L	Ischemic	Right MCA	1
1198	36	67	Black	M	75	R/R	Hemorrhagic	Left MCA	60
1248	36	39	White	M	30	R/L	Hemorrhagic	Right MCA	6
1195	42	68	Black	M	81	R/R	Ischemic	Left MCA (BG/IC)	0.5
1318	44	54	Asian	M	8	R/R	Ischemic	Left MCA	8
1228	56	44	Black	F	54	R/L	Ischemic	Right MCA	6
1280	57	62	White	M	25	R/L	Hemorrhagic	Right MCA	3
1291	58	58	American Indian	M	20	R/R	N/A	Left MCA	2
1317	58	59	Black	M	24	R/L	Ischemic	Right MCA	7

*N* = 13	38.8 (15.4)	52 (14)	Diverse ethnicities	9M/4F	46.4 (36.5)	5 R hemi/8 L hemi	5 Hemorrhage 5 Ischemia	Mostly MCA territory	8.5 (15.7)

*FMS, Fugl-Meyer Scale; OT, Occupational therapy, M, Male; F, Female; R, Right; L, Left; MCA, Middle Cerebral Artery*.

### Study Design and Intervention

We used a quasi-experimental mixed-method pre-test post-test design with 1-year follow up. Subjects received the 45-min intervention twice a week for 6 weeks in groups of three for a total of 12 sessions. One extra make-up session was provided for missed sessions due to holidays or illness. One music therapist (MT) provided the musical framework while playing the piano, while the occupational therapist (OT) and second MT facilitated subjects’ instrument playing. The therapists used hand-over-hand assistance or demonstration to encourage repetitive isolated movements of the affected upper limb while they played various musical instruments (Table 2). Abnormal compensatory movement patterns were discouraged by selecting appropriate instruments as described below. The group sessions were organized into introduction, interactive live music making and wrap-up intervals. The 5-min introduction consisted of the OT leading musically supported stretches of the trunk and upper limb, and a focus group discussion about the experience of living with stroke. The subjects were encouraged to openly share their feelings about their stroke with respect to physical, emotional, and/or psychosocial challenges and their overall well-being and quality of life. The 35-min music making consisted of improvised live music, with the MT and all subjects playing a variety of instruments, such as drums, bells, shakers, mallets, chimes, piano and harp with their affected upper limb as detailed below. Short breaks were provided as needed to change musical instruments, or to rest at the end of a “piece of music”. The therapists provided hand-over-hand support when subjects were particularly challenged, to maintain the flow of the music and facilitate engagement. Hand-over-hand support included the MT or OT grasping a subject’s affected upper limb and helping the subject to move through the range of motion needed to play the instrument. Specific attention was given to ensure that the subject’s movement was successful in creating a sound that contributed to the music, both with enough volume to be heard and with synchronization to the rhythm and, when applicable, to the melodic elements of the music. The instruments were adapted to make it easier for the subjects to use them. For example, instrument handles were enlarged using foam or rubber grips, Coban wrap was used to reinforce grasp, and devices such as adaptive picks for the harp or guitar were used. The orientation of the instrument was altered to enable playing (e.g., having the xylophone closer to the subject or at a different angle allowed for easier movements), and the subjects were encouraged to use both hands when they were unable to play the instrument with the affected hand alone (e.g., when using the maracas). When open chain movements were not possible to perform, closed-chain movements were used (e.g., holding the cabasa and rolling it on the thigh). In addition, the MT provided musical support to reflect the effort and expression of the subjects by adjusting the accompaniment. For example, dissonance was used to reflect physical exertion, high melodic registers were used to help extend arm reach, and the style of music was adapted to the mood of subjects while playing. The pulse and flow of the music was also adjusted to encourage smoother movements. When fatigue or use of compensatory strategies was noted, the MT reduced the intensity of the music or stopped the music for a period of rest. The 5-min wrap up involved final thoughts and feedback on the sessions from each of the subjects. The group discussions encouraged social participation as part of the intervention, and enabled the subjects to provide feedback on how the intervention affected them. All sessions were video-recorded for qualitative analysis.

**Table 2:** **Movements performed during MULT-I**.

Movement	Musical activity
Shoulder external rotation/internal rotation	1. Hold ***maraca*** in both hands with elbows at side, internally and externally rotate both shoulders together.
	2. Hold ***mallet*** in affected hand, reach to side repeatedly by externally and internally rotating shoulder to hit ***drum***.
	3. Hold ***tambourine*** with one hand, straighten the elbow, hit the tambourine with other hand repeatedly by internally and externally rotating at the shoulder; switch hands.
Shoulder flexion/extension	1. Hold ***maraca*** in both hands, reach up and down by flexing and extending at the shoulder.
	2. Hold ***mallet*** in affected hand, reach forward by flexing the shoulder to hit ***vertical bells***.
	3. Hold ***wind chime stand*** with the affected hand, push back and forth by extending and flexing at the shoulder.
Elbow flexion/extension	1. Hold ***mallet*** in both hands, hit ***xylophone or drum*** repeatedly by extending and flexing at the elbow.
	2. Hold ***horn*** with affected hand, bring to mouth to ***blow*** then bring down by flexing and extending at the elbow.
	3. Hold ***cabasa*** with affected hand, slide down leg and back by extending and flexing at the elbow.
Forearm supination/pronation	1. Hold ***maraca*** in affected hand with the elbow at side, then supinate and pronate forearm repeatedly.
	2. Hold ***rain stick*** with both hands, unaffected forearm supinated and affected forearm pronated; pronate unaffected forearm twisting affected forearm into supination, repeat.
Wrist flexion/extension and ulnar/radial deviation	1. Hold ***maracas*** in both hands, move up and down using wrist extension-flexion or radial-ulnar deviation.
	2. Hold ***hand chimes*** in affected hand, move briskly using wrist extension-flexion or radial-ulnar deviation.
	3. Hold ***rain stick*** with both hands, extend and flex both wrists repeatedly.
Hand grasp/release	1. Grasp similarly sized ***musical instruments*** with one hand, release to other hand; alternate hands.
	2. Grasp corn kernels with affected hand, release onto ***steel drum*.**
Finger individuation	1. Press keys on a ***piano*.**
	2. Pluck strings on ***harp or guitar*.**

Instrument selection was based on subjects’ preferences, as well as their abilities and deficits. For example, subjects with some finger movement but decreased fine motor coordination played the piano or the harp, whereas subjects with decreased hand function but adequate proximal arm movement played the drums. As several instruments can support similar movement goals, instruments that complemented one another in terms of tonality, timbre, volume and register were selected. The MULT-I intervention employed the Nordoff-Robbins approach to music therapy, which focuses on offering each group member an opportunity to feel successful and personally fulfilled in their experience of making music (Nordoff and Robbins, 2007/1977). This philosophy also influenced choice of instrument as it was important that each group member could be heard, and that individuals playing a melodic instrument would easily find notes fitting within the tonal structure of the music. Adaptations were made to the instruments to fit within the overall musical framework. For example, tone bars were added or removed from the xylophone to fit within the tonal framework of the music, and mallets were adjusted to change the volume of the instrument (yarn or felt tips were used to dampen the sound, and wooden or rubber tips were used to enhance the sound). Adaptations were also made to instruments to enable subjects to play their selected instrument successfully, as described above. Engagement in MULT-I required individuals to listen attentively to others, generate appropriately timed movements of sufficient velocity to produce sound, and sustain music making. The therapeutic goals were to enhance emotional awareness and expression, motivate spontaneous creative movement and interaction within the context of the intervention, and promote interpersonal communication, and sense of belonging to the group. Subjects were encouraged to bring in music that was meaningful to them to play during the sessions, to create a shared experience with personal investment in the music-making.

### Assessments

Clinical assessments were administered before and after the 6-week intervention and at 1-year follow up by a researcher who was not involved in the intervention. The primary outcome was reduction in motor impairment on the Fugl-Meyer Scale (FMS; Fugl-Meyer et al., 1975). This is a standard, validated, and widely-used scale of motor impairment post-stroke. It consists of 33 tasks, each of which is scored on a 3-point scale (0 = unable to perform, 1 = partially perform, 2 = faultless performance); the maximum score for the upper extremity is 66. The FMS score reflects the degree to which joint movements can be isolated. Secondary outcomes included reduction of sensory impairment (on the two-point discrimination test) and activity limitation (Modified Rankin Scale, MRS), and increase in well-being (World Health Organization well-being index) and participation post- stroke (Stroke Impact Scale (SIS) for activities of daily living and participation subscales, respectively). The two-point discrimination test (Mackinnon and Dellon, 1985) is a sensitive test for tactile sensibility, and has been shown to be predictive for upper limb dexterity after stroke (Meyer et al., 2014). The MRS (Rankin, 1957) is a six-level outcome scale to assess limitation in mobility and activities of daily living using a structured interview (Wilson et al., 2002). It has excellent inter-rater reliability (Wolfe et al., 1991) and criterion validity (Kwon et al., 2004). Well-being was measured using the World Health Organization (Five) well-being index (Tibaek et al., 2011). The SIS assesses stroke specific quality of life in eight domains (physical problems, memory and thinking, feelings and emotions, communication, activities of daily living, community mobility, use of the hand, and participation). The SIS has excellent test/re-test reliability, internal consistency and responsiveness (Duncan et al., 1999).

Kinematic data during wrist flexion/extension were also collected using a custom-made wrist independence trainer (WIT) pre- and post-intervention, but not at 1-year follow up (Aluru et al., 2014). The device was designed to limit movement to the wrist in the sagittal plane by stabilizing the forearm and arm on a platform using straps; it therefore discouraged compensatory movements. The height of the table was maintained across all subjects, with the front edge of the WIT aligned with the table. The height of the chair was adjusted for each subject to keep their shoulders level, trunk in proper alignment, and elbows at approximately 135° of extension. The arm rests on the WIT were adjusted to keep the forearms shoulder distance apart. Electromagnetic motion sensors (trakSTAR, Ascension Technology Corporation, Shelburne, VT, USA) affixed to the forearm and hand measured wrist kinematics. The data were captured using The Motion Monitor (Innovative Sports Training Inc., Chicago, IL, USA), and analysis was performed offline using Spike 2 (Cambridge Electronic Design, Cambridge, England). At the start position, the hand grasped the handle of the WIT and the wrist was in full flexion. The subject was instructed to perform as many wrist extension/flexion movements as possible within a 10-s period; this constituted one trial. The trials were performed first with the affected hand (unimanual), then with both hands (bimanual) in an alternating manner for a total of 11 trials: six trials with the affected hand interspersed with five trials with both hands. Transfer of learning from bimanual-to-unimanual trials was examined by plotting the range-of-motion of the affected wrist across the six trials with the affected hand (Aluru et al., 2014).

During the MULT-I intervention, qualitative information regarding the experience of life with stroke and reactions to the treatment intervention was obtained by video recordings of the sessions. While the treating OT and MTs initiated conversations on these topics, they promoted an open-ended discussion among the participants. The sessions were transcribed through the process of indexing (Aigen, 1993; Guerrero et al., 2014), which involves a detailed time-based index of the events of each session with transcription of both verbal and musical exchanges.

### Statistical Analysis

The General Linear Model (GENLIN) in IBM SPSS Statistics version 23 (IBM Corp., Armonk, NY, USA), was used to perform repeated measures analysis of variance (ANOVA) to test for differences in the means between pre-intervention, immediate post-intervention, and 1-year follow up scores, as the data were normally distributed. The analyses on the primary outcome measure (Fugl-Meyer scores) and secondary outcome measures (two-point discrimination, MRS, WHO well-being index and SIS subscales) included only subjects who completed all three assessments. Differences between the assessments were computed using the Wald Chi-square test. The reliability estimates (Cronbach’s alpha) for this sample were: FMS (0.954), static Two-point Discrimination test (0.879), WHO well-being index (0.816), and SIS activities of daily living (0.759) and participation (0.855) subscales. Exploratory analyses were conducted using repeated measures analysis of covariance (ANCOVA) to examine change in Fugl-Meyer Score over time by functional status, defined as the maximum extent of wrist extension pre-intervention: low-functioning subjects had <15° of wrist extension, whereas high-functioning subjects had >30° of wrist extension when using the WIT (Aluru et al., 2014). For these analyses, all subjects were included. The EM algorithm (Dempster et al., 1977) was used to impute missing data. The wrist kinematic data of the affected hand were also analyzed using ANCOVA to examine maximum change in wrist extension with bimanual-to-unimanual learning from pre- to post-intervention by functional status. Pearson’s correlation was performed to determine the relationship between pre-intervention Fugl-Meyer scores and maximum change in wrist extension. Qualitative analysis of focus group discussions recorded during the sessions was performed by compiling the indexes for the sessions, analyzing the content, and sorting it into emergent categories (by Nina Guerrero and Daniel Geller; Guerrero et al., 2014). The content was further analyzed in the context of the quantitative data to explore plausible explanations for the results obtained.

## Results

Sixteen subjects were enrolled; of these, thirteen subjects completed the intervention, and ten returned for follow up at 1 year. Two subjects dropped out in the first week due to pre-existing pain syndromes: one had a history of rheumatoid arthritis and the other had neuropathic pain in the upper limb. One subject participated in the sessions but fractured the wrist on his affected hand as a result of an unrelated fall prior to the post-intervention assessments. These subjects were not included in the analyses.

**Figure 1:**
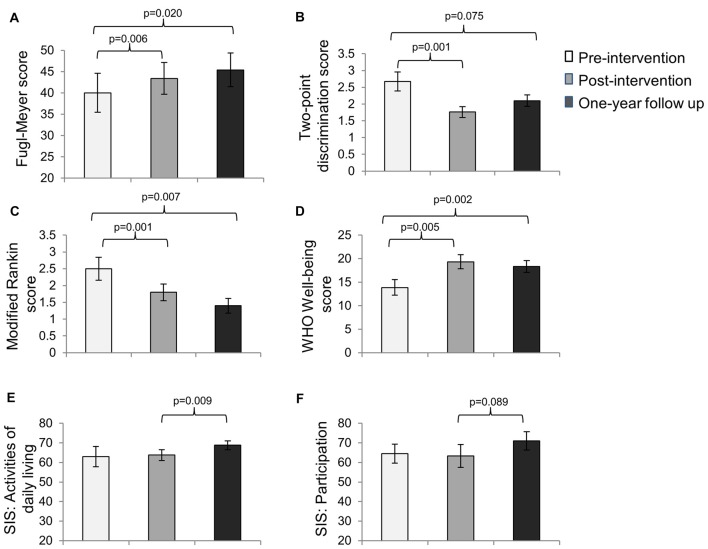
**Mean scores (±SE) on the (A) Fugl-Meyer Scale (FMS; *n* = 10), (B) Two-point Discrimination test (*n* = 7), (C) Modified Rankin Scale (MRS; *n* = 10), (D) the WHO Well-being scale (*n* = 9), (E) the activities of daily living subscale of the Stroke Impact Scale (SIS; *n* = 10) and (F) the participation subscale of the SIS (*n* = 10) at pre-intervention, immediate post-intervention and 1-year follow up assessments**.

For subjects who completed all three assessments, based on the repeated measures ANOVA, significant improvements in motor impairment were noted on the FMS (overall *p* = 0.021), sensory impairment on the two-point discrimination test (overall *p* = 0.002), disability on the MRS (overall *p* = 0.002), and well-being on the WHO well-being scale (overall *p* = 0.003) immediately post-intervention and were retained from pre-intervention to 1-year follow up (Figures 1A–D). There were no significant differences between post-intervention and 1-year follow-up.

Interestingly, the SIS scores for activities of daily living did not change significantly from pre-intervention to post-intervention (*p* = 0.79); however they increased significantly from post-intervention to 1-year follow up. Similarly, the SIS scores for participation did not change significantly from pre-intervention to post-intervention (*p* = 0.84), but showed a trend toward significance from post-intervention to 1-year follow up (Figures 1E,F).

**Figure 2:**
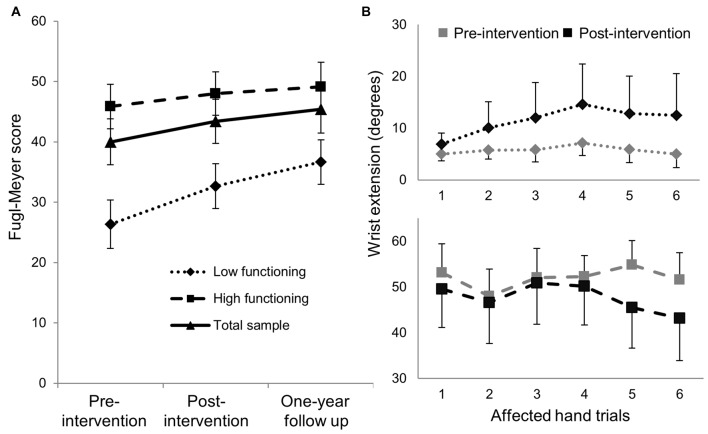
**(A)** Mean (±SE) Fugl-Meyer scores in low-functioning subjects (*n* = 5) showed greater improvement compared with high-functioning subjects (*n* = 8). **(B)** The low-functioning subjects (active wrist range-of-motion <15°) showed better bimanual-to-unimanual learning across trials with the affected hand post-intervention than the high-functioning subjects (active wrist range-of-motion >30°).

Exploratory analyses using repeated measures ANCOVA revealed a significant increase in the Fugl-Meyer scores over time based on functional status defined by degree of active wrist range-of-motion pre-intervention (*p* = 0.007; Table 3). Low-functioning subjects, who had less than 15° of active wrist extension pre-intervention (*n* = 5), showed higher gains on the FMS post-intervention and at 1-year follow up compared with high-functioning (*n* = 8) subjects who had greater than 30° of active wrist extension (Figure 2A). Maximum change in wrist extension with bimanual-to-unimanual learning was significantly different between the low-functioning and high-functioning groups pre-intervention (*p* = 0.003). Low-functioning subjects showed a trend toward significance for improvement in maximum wrist extension with bimanual-to-unimanual learning post-intervention compared to the high-functioning subjects (*p* = 0.06; Figure 2B). The study was powered to detect only large effects. Pre-intervention Fugl-Meyer scores were positively correlated with the pre-intervention maximum change in wrist extension with bimanual-to-unimanual learning (*r* = 0.85), but were negatively correlated with the change in maximum wrist extension from pre- to post-intervention (*r* = −0.52).

**Table 3:** **Predicting change in Fugl-Meyer Score over time by functional status**.

	Estimate	Std. Error	Wald Chi-Square	*p*-value
Intercept	24.99	4.02	38.602	<0.001
Fugl-Meyer Score	5.83	1.05	31.009	<0.001
Functional Status	22.95	5.45	17.700	<0.001
Fugl-Meyer Score by	−4.91	1.82	7.244	0.007
Functional Status

The qualitative data obtained from the therapy sessions were explored for plausible explanations for the quantitative results obtained, particularly with regard to feelings of body ownership and agency of action. Qualitative data were also explored for moments when subjects shared their challenges regarding their overall recovery process and participation in the intervention. Sharing challenges was considered a significant aspect of the intervention, as these challenges could then be addressed, through physical, emotional and social support. Five main themes were noted: challenges with feelings of ownership of the impaired arm, increased feelings of ownership of the impaired arm with MULT-I, more spontaneous movement, enhanced emotional engagement, and challenges during the intervention. The statements below were captured during group discussions, organized by session. Statements from all 13 subjects who completed the intervention, including both low- and high-functioning subjects, are represented under each theme.

Theme 1: Challenges with feelings of ownership of the impaired arm

1228 (session 3) *“I forget my [affected] arm doesn’t do what I want it to do.”*
1257 (session 3) *“I need to improve. I totally forget about the left side. I put stuff [under my left arm] and forget it.”*
1195 (session 3) *“My brain has to tell my arm it’s there.”*
1248 (session 7) *“It’s hard to get my arm involved in the things I do every day.”*
1280 (session 7) *“I can’t get my arm to do what my brain tells it to do.”*
1257 (session 9) *“I used to be the kind of person who always helped—now people do things for me.”*
1257 (session 11) *“This is really sad…they tell me eventually I will feel [my arm]. But every morning I wake up, and it’s still the same.”*

Theme 2: Increased feelings of ownership of the impaired arm with MULT-I

1300 (session 2) *“I felt a lot more strength in my left [affected] arm toward the end of the music.”*
1198 (session 7) *“I feel relief, to be able to move the arm.”*
1257 (session 8) *“I’m much more conscious of my left arm now—certainly in the sessions, but also throughout the day. I at least try to use it.”*
1228 (session 8) “*Music stimulates lots of different movements, more movements than usual and different kinds. People make comments about my [affected] arm. They say ‘you’re moving it better’.”*
1237 (session *11)“This morning, it was so cool…I was lying down and could totally feel my left [affected] arm, and I could feel the weight of it against my body*.”

Theme 3: More spontaneous movement

1195 (session 2) *“I was cooking and I spontaneously reached out with my right* *[affected]* *arm.”*
1198 (session 2) *“My wife observed that I put on my jacket by myself.”*
1201 (session 7) *“I can now open my hand supporting my weight on the bed.”*
1237 (session 8)*“I started taking the train, and gave up using my cane.”*
1237 (session 10) *“I started dancing with my son to his favorite music.”*

Theme 4: Enhanced emotional engagement

1198 (session 1) *“I feel the music!”*
1317 (session 1) *“Music calms the soul.”*
1228 (session 2) *“This is fun.”*
1291 (session 2) *“I feel more joy.”*
1198 (session 2) “*[The intervention]* *allows participants to have healthy interaction with others in similar circumstances, and is just plain fun!”*
1237 (session 8) *“I get lost in the music; I could do this all day.”*
1228 (session 9) *“There are still certain movements…that I hate to do because they give me that sickly feeling…[but] the music lets us ride it out.”*
1257 (session 9) *“I’ve been through many different therapies, and with no disrespect to all the therapists I’ve had, this is more fun! More interesting and varied.”*
1318 (session 13) “*Music makes movement less tiring and less boring.”*
1280 (session 13) *“We’re creating something together; the whole is greater than the sum of its parts.”*

Theme 5: Challenges during the intervention

1291 (session 8) *“My arm is going crazy….All this isn’t helping with my arm! It’s very difficult, uncomfortable.”*
1237 (session 8) “*With all of the sensations I’m getting in my left arm, it triggers my spasticity. My arm is tingling.”*
1243 (session 8) *“The length of treatment is too short.”*
1228 (session 9) *“I hate it…it’s boring…the tone chime swings back and hits my left knuckles…it hurts!”*
1291 (session 11) *“I’m having trouble with my voice… I sound retarded.”*

## Discussion

This proof-of-concept study tested the hypothesis that an enriched collaborative group music-making intervention, Music Upper Limb Therapy-Integrated (MULT-I), that combined music therapy with occupational therapy to support physical effort, psychological well-being and social participation simultaneously, will lead to reduced upper limb motor impairment (primary outcome), and reduced sensory impairment and activity limitation along with increased well-being and participation (secondary outcomes) post-intervention, and that the improvement would persist at 1-year follow up. The results support our hypotheses. Interestingly, activities of daily living and social participation improved only from post-intervention to 1-year follow up. Furthermore, low-functioning subjects showed greater improvement on motor impairment with this intervention compared with high-functioning subjects. Qualitative analyses of group discussions that were part of the intervention, suggest that the subjects experienced challenges with feelings of ownership of the impaired arm, but that the MULT-I intervention helped increase their feelings of ownership of the impaired arm, promoted spontaneous movement, and enhanced emotional engagement, despite challenges during the intervention. These results are discussed below.

### Impacting Disability Across Multiple Domains with MULT-I

The goal of rehabilitation is to impact disability in all three domains of the International Classification of Functioning, Disability and Health’s (ICF) model (Geyh et al., 2004)—impairment, activity and participation. The relationships among these domains are complex and influenced by many factors which are difficult to address with any single intervention. Combination therapies or interventions that are designed to address multiple facets of rehabilitation simultaneously may be one solution. The MULT-I intervention offers several advantages over traditional therapy that can contribute to enhanced network connectivity conducive to post-stroke recovery. Music modulates activity in a broad bilateral network of mesolimbic structures involved in processing emotions and reward information (Menon and Levitin, 2005). The recruitment of limbic circuits, for example during creative musical activity (Bashwiner et al., 2016), may facilitate motor recovery after stroke (Marshall et al., 2009). Repetitive sensory stimulation from the use of percussion instruments may enhance functional connectivity in the sensorimotor network (Freyer et al., 2012). In addition, moving in synchrony to an auditory beat enhances the perception of beat timing (Manning and Schutz, 2013), leading to coordination of perception and action for sensorimotor integration. Auditory-motor coupling during rhythmic tasks activates a cortico-subcortical network including the auditory cortex, putamen, supplementary motor area and the premotor cortex, engaged in the analysis of temporal sequences, prediction of beat and beat generation (Grahn and Rowe, 2009; Rodriguez-Fornells et al., 2012). Furthermore, music can enhance social cognition (Koelsch, 2014, 2015). Taken together with the present findings, these data suggest that the MULT-I intervention created an enriched environment that provided simultaneous physical, psychological and social engagement leading to improvement in all three domains of disability over the long-term.

### Longer-Term Improvement in Activities of Daily Living and Participation

Our results showed no significant improvement in activities of daily living and participation subscales of the SIS immediately post-intervention, but the improvement was significant (or trended towards it) between post-intervention and 1-year follow up. The SIS is a valid and reliable outcome measure with robust psychometric characteristics to measure the impact of stroke on activities of daily living and participation (Duncan et al., 2003), and the participation subscale effectively captures social well-being and quality of life in patients with stroke (Lai et al., 2003). However, the responsiveness to change is affected by stroke severity and time since stroke, and changes in quality of life are typically measured at 3-month intervals with rehabilitation interventions (Duncan et al., 1999). The 6-week interval between pre- and post-intervention in this study may have been insufficient for changes in quality of life to take place. Overall, the improvement in upper limb motor and sensory impairment, activity limitation, and well-being from pre- to post-intervention, and the improvement in activities of daily living and participation from post-intervention to 1-year follow up, suggest that the MULT-I intervention is promising to effect long-term post-stroke recovery.

### Functional Status Predicts Improvement with MULT-I

Rehabilitation trials typically show greater improvement in subjects with higher pre-intervention Fugl-Meyer scores, compared to those with lower Fugl-Meyer scores (Gebruers et al., 2014), because the higher scores reflect lower stroke severity and the availability of neural substrates to mediate recovery. Interestingly, in this study, low-functioning subjects, who had limited movement in their wrist and hand, showed a significantly greater reduction in motor impairment than the higher- functioning subjects who had more movement to begin with, but were still far from complete recovery. Furthermore, the lower the pre-intervention Fugl-Meyer score, the greater was the increase in maximum wrist extension from pre- to post-intervention with bimanual-to-unimanual learning. We have previously suggested that subjects with predominant paresis, i.e., with limited active wrist range of motion and low Fugl-Meyer scores, may be particularly responsive to rhythm and music (Aluru et al., 2014). Rhythmic auditory stimulation has been shown to produce more efficient motor unit synchronization (Thaut et al., 1999), which can directly facilitate movement. It also excites a bilaterally distributed sensorimotor network (Pollok et al., 2005; Serrien, 2008) and increases excitability of spinal motor neurons via the reticulospinal pathway (Paltsev and Elner, 1967; Rossignol and Jones, 1976), that can increase co-activation across both the agonist and antagonist muscles. While any muscle activation may contribute to increased movement in low-functioning individuals, as previously shown (Aluru et al., 2014), and exemplified by subject #1237 in this cohort (see statements under Theme 5), excessive muscle co-activation in higher-functioning individuals (e.g., subject #1291 (session 8) under Theme 5) may actually reinforce abnormal synergy patterns, produce discomfort, and decrease movement. Hence, rhythmic auditory engagement in sensorimotor tasks may be particularly beneficial to facilitate movement in low-functioning individuals.

### Adjusting to a New Identity Post-Stroke

Stroke is experienced as a sudden and overwhelming disruption, impeding engagement in previously easy tasks, and altering stroke survivors’ sense of self (Salter et al., 2008). Experiences that support re-connection with a new body and positive social interactions are important to the recovery process and for re-establishing a positive sense of self. Activities for which there is no perceived consequence for failure support the process of re-establishing a connection with the body after a stroke (Guidetti et al., 2007). Music-making activities, particularly involving rhythmic use of the body in conjunction with others, provide an opportunity to reconnect with the body. The temporal structure of musical rhythm facilitates sensorimotor synchronization (Merker et al., 2009; Ravignani et al., 2014). Perceiving rhythm (Maes et al., 2014) and observing rhythmic movements (Kirsch and Cross, 2015) enable the formation of internal representations of familiar movement kinematics (Stadler et al., 2012). Furthermore, rhythmic music-making activities, as in the MULT-I intervention (Table 2), are not part of most participants’ activities of daily living, which reduces the perceived consequences of failure.

Additionally, different instruments require different degrees of bodily engagement and control. Thus, instrument selection is important to help the stroke survivor feel successful in his or her ability to make music effectively, and receive positive feedback through the sounds created and the physical sensation of playing. Instrument selection was performed collaboratively by the subject, MT and OT. Moreover, the musical framework created by the therapists highlighted even the smallest movement or sound made by a subject. The qualitative data presented under Themes 2 and 3 suggest that the MULT-I intervention was successful in helping subjects reconnect with their body, increase feelings of ownership of the impaired arm, and move more spontaneously.

Group music therapy has been shown to improve social interaction among stroke survivors (Nayak et al., 2000). The live interactive music-making in MULT-I created a psychologically safe environment that reflected the emotional expressions and physical activities of the group. Through music, individual members expressed a full range of emotions and connected with other members in the group. The qualitative data presented under Theme 4 suggest that the group music-making during MULT-I successfully enhanced emotional engagement. Subjects not only reported feeling a part of creating something together, but were also able to explore difficult feelings of loss, grief and frustration (under Themes 1 and 5), and emerged from the experience with an improved sense of well-being. Taken together, the improved awareness of the affected limb combined with enhanced emotional engagement suggest that the MULT-I intervention supported the creation of a positive sense of self post-stroke.

### Integrating the Benefits of Music Therapy to Enhance Stroke Rehabilitation

Current practice in music therapy and stroke rehabilitation includes multiple theoretical perspectives (Magee and Baker, 2009). Neurologic Music Therapy harnesses the benefits of auditory-motor coupling to facilitate gait training and upper limb rehabilitation (Thaut et al., 2007; Malcolm et al., 2009). Other music therapy methods facilitate self-expression, connection with others, and emotional well-being through instrumental improvisation and group singing (Magee and Baker, 2009). Music-listening, alone, has been shown to improve cognition and mood among stroke survivors (Särkämö et al., 2008). There is a need to develop a music-based intervention that effectively integrates these benefits of music therapy into a comprehensive approach for stroke rehabilitation.

The MULT-I intervention leverages the benefits of auditory-motor coupling, while allowing flexibility in the music-making process through selection of instrument, musical style, and improvisation to support the psychosocial aspects of stroke rehabilitation, which contribute significantly to overall rehabilitation outcome (Ostir et al., 2001). The collaboration between music therapy and occupational therapy further supports functional goal attainment within a group music-making process. In addition, enjoyable tasks motivate individuals to engage in movement, and provide a positively reinforcing experience for post-stroke re-learning (Sabini et al., 2013). The results of this study suggest that it is feasible to integrate the physical, psychological and social benefits of music into a single effective intervention for stroke rehabilitation.

### Limitations and Conclusions

This quasi-experimental study had a pre-test post-test design and a small sample size. There was no control group because the purpose of the study was to determine the feasibility of the enriched collaborative MULT-I intervention in facilitating long-term upper limb recovery across multiple domains of disability. This study is innovative in many respects. First, the intervention is based on the synergistic effects of physical, psychological and social rhythmic entrainment which can help create both a sense of ownership and agency with regard to the impaired limb. Second, the study evaluates the feasibility of a collaborative intervention for post-stroke recovery which involves not only inter-disciplinary collaboration between MTs and OTs but also collaboration among patients and therapists. Such collaborative interventions could potentially increase cost-effective access to rehabilitation. Third, our results demonstrate that it is possible to impact all three domains of disability, i.e., impairment, activity and participation limitations, with a single relatively short-term intervention with long-lasting results. Finally, this study provides preliminary evidence that this intervention may be more effective in a lower functioning subgroup of patients. This is important for resource allocation to target the right patients and obtain the best outcomes. Taken together, our results suggest that the MULT-I intervention may constitute a practical model of an enriched environment for post-stroke rehabilitation even in the chronic stage post stroke. This holds significant implications for further research and clinical use of enriched interventions for post-stroke rehabilitation. The next step is to perform a larger randomized controlled study to confirm the results. We are now testing the translatability of the MULT-I intervention with larger group sizes and fewer therapists, and in low-resource settings, for more cost-effective resource utilization.

## Author Contributions

PR and AT: conception and study design. DG, NG, VA, AT: study execution. JPE, JAT, GO, VA, PR: data analysis and interpretation. PR, DG, NG, AP, VA: manuscript drafting.

## Funding

This work was supported by the NYU Steinhardt Community Collaborative Challenge and the Arthur Flagler Fultz Research Award from the American Music Therapy Association to PR and AT, and in part by the NYU CTSA grant UL1TR000038 from the National Center for Advancing Translational Sciences (NCATS) and U54NS081765.

## Conflict of Interest Statement

The authors declare that the research was conducted in the absence of any commercial or financial relationships that could be construed as a potential conflict of interest.
